# Numerical Assessment of Elliptical Pore Orientation and Eccentricity Effects on Charge Transport in Anisotropic Functional Membranes

**DOI:** 10.3390/membranes15120370

**Published:** 2025-12-02

**Authors:** Carlos Pacheco, Alfonso Navarro, Enrique Escobedo, Romeli Barbosa

**Affiliations:** Centro de Investigación Científica de Yucatán, Parque Científico y Tecnológico de Yucatán, Carretera Sierra Papacal–Chuburna Puerto, km 5, Sierra Papacal, Merida 97302, Yucatan, Mexico; dimathidrogeno@cicy.mx (C.P.);

**Keywords:** porous membranes, anisotropy, pore eccentricity, finite-volume method, effective conductivity, charge transport, membrane design, energy conversion

## Abstract

The transport efficiency of anisotropic functional membranes is largely dictated by the geometry and orientation of their internal pores. In this study, a numerical finite-volume framework was developed to evaluate how elliptical pore eccentricity (εcc) and orientation influence charge transport and effective conductivity ($e_{k}$) within two-dimensional porous membrane microstructures. Canonical stochastic domains with controlled porosity were generated, considering parallel and perpendicular aligned configurations of the major pore axis relative to the imposed potential gradient. Results demonstrated a strong orientation dependence: under perpendicular alignment, the effective conductivity decreased by up to 70% as *εcc* increased from 0.5 to 0.999, while parallel alignment maintained at $e_{k}$ > 0.8 even for highly elongated pores. The aspect ratio (b/a) was identified as a secondary geometric modulator producing opposite conductivity trends depending on orientation. Through isotropy-error analysis, a critical morphological threshold at εcc ≈ 0.9 was found, indicating the onset of structural anisotropy and loss of isotropic transport. These results establish a quantitative structure–property relationship linking pore geometry to macroscopic transport performance. The proposed stochastic FVM-based approach provides a generalizable and computationally efficient tool for the design and optimization of anisotropic porous membranes used in electrochemical and energy-conversion devices.

## 1. Introduction

The current energy transition demands more efficient and sustainable technologies for energy conversion, storage and utilization. In this context, hydrogen ($H_{2}$) is emerging as a key energy carrier. Using proton exchange membrane (PEM) technologies, $H_{2}$ can be generated through intermittent power supply from wind and/or photovoltaic systems by PEM water electrolyzers [1,2], compressed by PEM electrochemical $H_{2}\;$compressors [3], and converted to constant electrical energy by PEM fuel cells [4]. These technologies, mainly composed of electrochemical cells, perform hydrogen transformation through membrane-based electrochemical systems where charge and mass transport govern overall efficiency.

The functional membrane (FM) in these electrochemical devices can be regarded as a stochastic heterogeneous material (SHM), characterized by its multiphase architecture and interconnected porous domains [5,6]. Within such porous microstructures, charge transport, ion conduction, and gas diffusion play a decisive role in energy conversion efficiency [7]. The microstructure of the membrane is composed of a solid conductive matrix interspersed with a network of transport pores that facilitate reactant access and product removal [6,7]. The morphology of these pores (their shape, size, and orientation) governs the continuity of conductive pathways and the effective transport properties. These aspects are quantified by effective transport coefficients (ETCs), which provide a macroscopic measure of the membrane’s global transport behavior [5,7].

On the other hand, during the assembly and operation process, the applied mechanical stress can induce deformations in the porous structure, transforming originally circular pores into elliptical geometries [8,9,10]. Understanding how porous membranes respond to such deformation is critical for predicting structural stability and transport durability [11]. Morphological evolution directly affects the effective electrical conductivity and defines the charge-transport efficiency ($e_{k}$) within the functional membrane [6,12]. A detailed analysis of ETCs and their variation with porosity and deformation is therefore essential to optimize the performance of membranes used in electrochemical hydrogen technologies [5,7]. This perspective supports the rational design of porous membranes with tailored transport characteristics to enhance both efficiency and durability [13,14].

In recent years, multiple investigations have employed digital reconstructions and numerical techniques to analyze the relationship between microstructural morphology and functional performance in porous membranes. For instance, some studies have used the finite volume method to analyze the effect of isotropy on the effective transport coefficient, demonstrating the sensitivity of macroscopic properties to microstructural organization [7,15,16]. Through synthetic imaging and computational modeling, aspects such as tortuosity, phase connectivity, percolation, and anisotropy have been explored, providing valuable insights to predict and improve the performance of membrane-based systems used in fuel cells, batteries, sensors, and energy devices [15,16]. These studies have been key to understanding membrane transport phenomena and their role in advanced energy materials [7,17].

One of the least explored geometric aspects in this context is pore eccentricity, defined as a measure of deviation from an ideal circular shape [9,10]. In elliptical pores, this property (expressed as the ratio between the semi-major and minor axes) can significantly influence how the electric flux or diffusive transport is distributed, affecting both the effective transport coefficients and overall charge-transport efficiency ($e_{k}$) [6,12]. This analysis is especially relevant for understanding transport behavior in anisotropic porous membranes and their impact on the performance of electrochemical energy systems [18,19].

Modeling the transport properties of such complex membrane microstructures presents significant computational challenges. While various numerical techniques, like the Finite Element Method (FEM) [20] and the Lattice Boltzmann Method (LBM) [21], have been widely employed for transport studies, multiscale approaches combining 3D microstructure reconstruction with the Finite Volume Method (FVM) have demonstrated high accuracy in predicting membrane transport coefficients [22]. Accurately capturing the coupled effects of stochastic pore distribution, geometric anisotropy, and phase connectivity remains an active research area [23]. Developing computationally efficient frameworks that correlate specific membrane morphological parameters, such as pore eccentricity, with macroscopic transport behavior, is essential for advancing the rational design of porous membranes.

This work presents a numerical and theoretical analysis of the effect of pore eccentricity (εcc) and orientation in anisotropic porous membranes. Using a combination of deterministic and stochastic numerical techniques, the influence of microstructural geometry on charge-transport efficiency ($e_{k}$) was systematically analyzed [7]. Two canonical configurations were evaluated: (1) perpendicular alignment of the major pore axis relative to the transport direction ($e_{k,perp}$) and (2) parallel alignment ($e_{k,parallel}$), providing quantitative insight into the anisotropic transport behavior of functional membranes.

## 2. Materials and Methods

**Figure 1:**
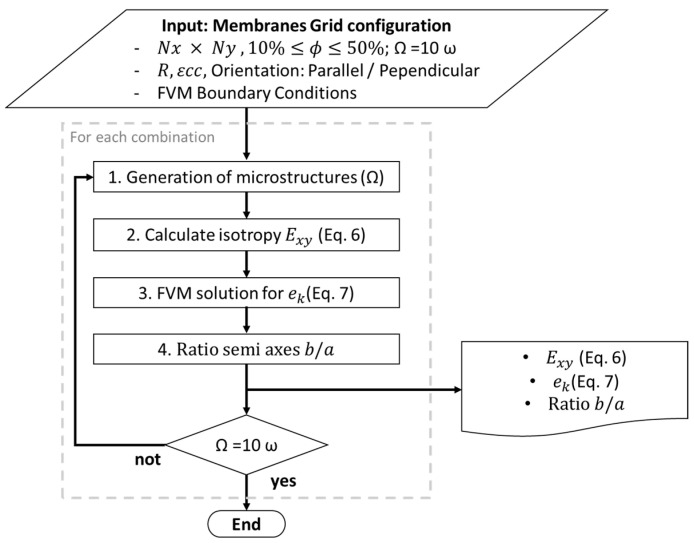
Flow chart of the generation, evaluation and simulation of the effect of pore eccentricity (εcc) on charge transport efficiency ($e_{k}$).

A proprietary computational algorithm was implemented in C++ (Dev-C++ 5.11) to investigate how εcc and orientation influence the $e_{k}$ of porous functional membranes. The methodology is divided into three main stages: (1) stochastic generation of canonical membrane microstructures, (2) evaluation of geometrical isotropy through two-point correlation functions, and (3) calculation of the $e_{k}$ using the FVM to solve the steady-state charge-continuity equation. The steps are described in the block diagram in Figure 1.

### 2.1. Generation of the Mesh of Canonical Microstructures

Random binary matrices ω*i*(*x*,*y*) were generated to represent two-phase membrane domains, where the solid phase corresponds to the conductive backbone and the void phase to porous transport channels.

A two-dimensional matrix of $N_{x}\times \;N_{y}\;$(400 × 400) pixels representing the simulation domain was generated. Each pixel was defined as solid (conductive material) or porous (insulating material). Nodes were randomly inserted according to a 1-point index function, until a target porosity *Φ* was reached, as described by Equations (1) and (2).

(1) $$\omega_{i}=\left\{R_{x_{i},y_{i}}\;|\;i=1,\;\ldots ,\;N_{x};\;j=1,\;\ldots ,\;N_{y}\right\}$$

(2) $$R_{x_{i},y_{i}}=\left\{\begin{array}{c} R_{x_{i+a},y_{i+b}}=1\;if\;random\;\leq seeds\;and\;\sum_{N_{x},\;N_{y}=0}^{N_{x}\times N_{y}}R\leq \phi \\ R_{x_{i+a},y_{i+b}}=0\;otherwise\; \end{array}\right.$$

The geometry of each elliptical pore is characterized by εcc, which is determined by its semi-major axis a and semi-minor axis b, according to Equations (3) and (4):

(3) $$a=\frac{R}{\sqrt[{4}]{\left({1-\varepsilon cc}^{2}\right)}}$$

(4) $$b=R{\left({1-\varepsilon cc}^{2}\right)}^{-1/4}$$

where R is the base radius of a circular pore. To assign a physical dimension to the matrix [24], it was established that 18 pixels correspond to 0.04 μm, so the spatial resolution is:

(5) $$Area=\frac{0.04\;\mu m}{18\;pixeles}=2.22\;\times \;10^{-3}\;\mu m/pixel.$$

The conservation of porosity during geometric deformation is an intentional idealization used to decouple shape-induced tortuosity from density-driven transport effects. Varying the semi-major and semi-minor axes at constant ϕ allows for isolating the impact of eccentricity and orientation on conductive path continuity without introducing the additional variability associated with pore collapse. In real membranes, compression alters ϕ through pore coalescence, necking and partial closure [25,26], which would confound the interpretation of the microstructural trends targeted here. This modeling choice therefore enables a controlled comparison of the geometric mechanisms that drive the observed anisotropy.

Thus, the full domain (400 pixels per side) represents a physical area of approximately 0.89 μm × 0.89 μm, corresponding to the characteristic pore-scale dimension observed in carbon-based functional membranes for energy and gas-transport applications (e.g., PEM electrolyzers, fuel cells).

Although the context of this work focused on carbon-based membranes used in fuel cells, the model presented is general in nature and its conclusions are applicable to other two-phase porous systems where charge transport is a dominant factor.

**Figure 2:**
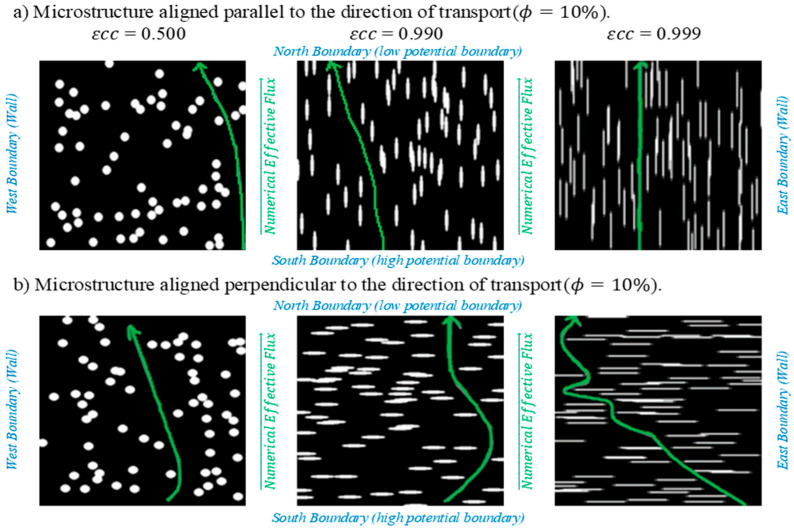
Binary images of stochastically generated porous membrane microstructures with different εcc (ϕ = 10%). The FVM simulations were performed by applying a potential difference between the north and south boundaries: (**a**) parallel orientation, (**b**) perpendicular orientation to the transport direction.

To assess the impact of pore orientation as a function of εcc, two canonical configurations were considered: (1) elliptical pores perpendicular to the imposed potential gradient, and (2) elliptical pores parallel to it. When εcc = 0, the structure corresponds to circular pores; as εcc → 1, the pores become increasingly elongated, altering tortuosity and connectivity within the membrane. Porosities in the range 10% ≤ ϕ ≤ 50% were analyzed, representing typical void fractions in porous membranes used for electrochemical and separation processes. Figure 2 shows representative images when ϕ = 10%, from left to right, the increase in *ε* is represented.

The eccentricity range εcc (0.5–0.9) corresponds to deformation levels commonly observed in carbon-based and PFSA membranes subjected to assembly compression or long-term operation [25,26,27]. These values represent geometries in which circular pores are progressively elongated while maintaining realistic shape distortions associated with load-induced collapse. In contrast, configurations with εcc → 1 were included as a numerical probe to isolate the onset of the geometric anisotropy and evaluate the worst-case tortuosity amplification in perpendicular alignment. These high-eccentricity limits are therefore not intended to represent realistic manufacturing conditions, but rather to explore the structural boundary of transport degradation.

In Figure 2, the blue text specifies the boundary conditions defined to solve the charge transport continuity. In each finite-volume mesh, a potential difference was imposed between the south (input) and north (output) boundaries to evaluate the effective flux [7]. Figure 2a shows a microstructure in which the elliptical pores are oriented parallel to the transport direction (from south to north), in this case ϕ = 10%. In Figure 2b, it can be seen that the elliptical pores are arranged perpendicular to the transport direction. Graphically, it can be observed that the parallel orientation (Figure 2a) allows for more direct and less tortuous trajectories for the electrical conduction from south to north. This definitely means a higher $e_{k}$. The opposite happens in the perpendicular orientation (Figure 2b), graphically, it was observed that the pores hinder the charge flow paths, increasing the tortuosity of the microstructure. In this work, we quantify numerically $e_{k}=f(\varepsilon cc,\phi ,\;R)$.

### 2.2. Isotropy Evaluation

The isotropy of a membrane microstructure can be characterized through the orientation of the tensor describing the transport phenomenon [28], or as a geometrical constraint conditioning the structure of the covariance matrix in a random field [29]. In this context, the relationship between the sum of covariance models in factor spaces and zonal isotropy, as well as the correspondence between geometric isotropy and isotropic covariance functions are particularly relevant [30]. It should be noted that geometric isotropy and zonal isotropy are intrinsically related, and their distinction is essential for an accurate characterization of microstructures.

Geometric isotropy is a robust tool for describing the behavior of microstructures, especially when high-fidelity models, such as high-resolution SEM images or stochastically reconstructed membrane domains, are available. One of the challenges lies in the identification and application of appropriate scaling factors, which represents a significant limitation in current modeling and research efforts. In this study, the two-point correlation function was used to determine the normalized autocovariance of the spatial distribution of the vertical axis $\chi_{j,y}^{*}(r)$ and the horizontal $\chi_{j,x}^{*}(r)$ [31]. The difference between the two functions was quantified by the mean square error $E_{xy}$, defined as:

(6) $$E_{xy}=\frac{1}{n}\sum {\left[\chi_{j,x}^{*}(r)\;-\;\chi_{j,y}^{*}(r)\right]}^{2}$$

where n is the total number of distances evaluated. In this definition, Exy→0 when εcc→0 (tendency to isotropic microstructure) and Exy→0.02 when εcc→1.00 (tendency to anisotropic microstructure). The normalized autocovariance was selected because it directly captures symmetry breaking in spatial distributions and correlates with direction-dependent transport. Alternative descriptors such as Minkowski functionals or path-length based tortuosity indices quantify complementary aspects of morphology but require independent numerical procedures and calibration to each microstructure family. Since this study focused on anisotropy emerging from controlled geometric perturbations, a single directional descriptor is sufficient to detect the transition from isotropic to structure-dominated regimes.

### 2.3. Charge Transport Efficiency ($e_{k}$)

The FVM was applied to the control-volume meshes generated in Section 2.1. Each node represents a local conductivity phase within the membrane domain. The FVM ensures strict conservation of the current flux, making it ideal for membrane transport simulations in both the 2D and 3D systems [32]. The normalization and generalization of the results is performed by applying the second law of thermodynamics, defining $e_{k}$ as the ratio between an effective (real) conductivity, over a nominal (ideal) conductivity, as defined by Equation (7):

(7) $$e_{k}=\;\frac{\Gamma_{eff}}{\Gamma_{M}}$$

where $\Gamma_{eff}$ corresponds to the overall effective electronic conductivity through the porous membrane and $\Gamma_{M}$ is the nominal conductivity.

The FVM was chosen for its robustness in handling complex geometries and conservation properties inherent to transport phenomena. This 2D model effectively captures the fundamental influence of pore orientation and eccentricity on membrane-scale transport behavior while maintaining high computational efficiency.

A grid-independence verification was performed based on a previously validated finite-volume framework applied to heterogeneous porous media [33]. In that study, domain refinement from 400 to 1200 pixels yielded variations below 2% in the effective transport coefficient while preserving the morphological transport trends. Following this criterion, the resolution of 400 × 400 pixels adopted here falls within the stable regime, in which numerical fluctuations remain significantly smaller than the orientation- and eccentricity-induced variations. Since the present work focused on relative conductivity trends rather than absolute predictive values, selecting a grid size inside the established stability window was sufficient to avoid mesh-dependent artifacts.

It should be emphasized that the present numerical framework operates within the continuum regime, in which local conductivity and volumetric flux conservation remain valid. When pore dimensions approach the sub-nanometer scale, transport mechanisms depart from classical diffusive–Ohmic behavior and become dominated by interfacial scattering, confinement effects, and hopping conduction. Under such conditions, assumptions of uniform material properties and FVM discretization cease to be valid, and alternative models (e.g., sorption–diffusion or molecular dynamics) are required to describe transport accurately [34,35].

The finite-volume solver iterates until the normalized residual of the continuity equation falls below 10^−6^, ensuring strict flux conservation. A Gauss–Seidel scheme with under-relaxation was adopted for stability, and convergence was reached within 3500–5200 iterations for all Ω configurations. No oscillatory behavior or divergence was observed, confirming that the observed variations in $e_{k}$ originate from geometric rather than numerical effects.

## 3. Results

**Figure 3:**
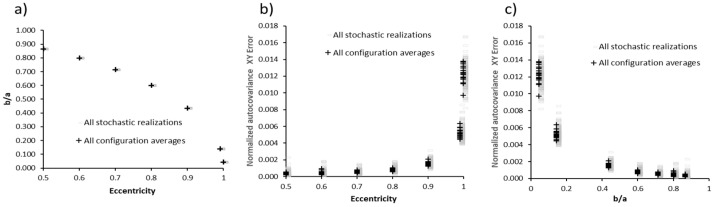
(**a**) Relationship between the semi-axes b/a as a function of εcc,showing the values of all stochastic realizations (gray) and the averages of each configuration (black); (**b**) Normalized autocorrelation error *XY* as a function of εcc. (**c**) Evolution of the normalized autocorrelation error *XY* as a function of b/a.

**Figure 4:**
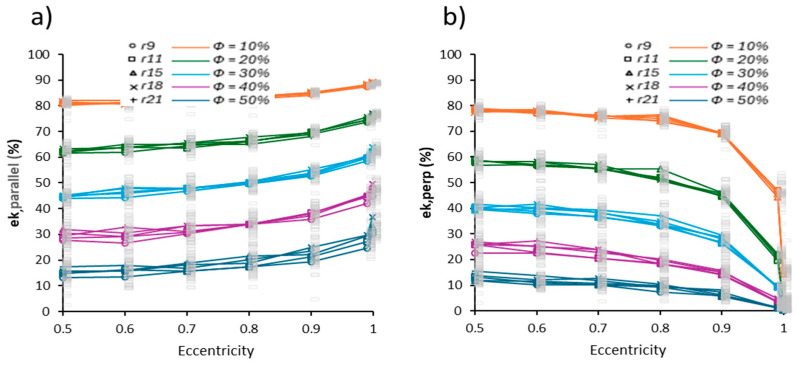
Charge transport efficiency $e_{k}$ as a function of eccentricity εcc. (**a**) For pores aligned parallel; (**b**) for pores aligned perpendicular to the direction of charge transport. Individual values of each stochastic realization ω (gray dots) are shown along with the average per configuration Ω (solid lines).

**Figure 5:**
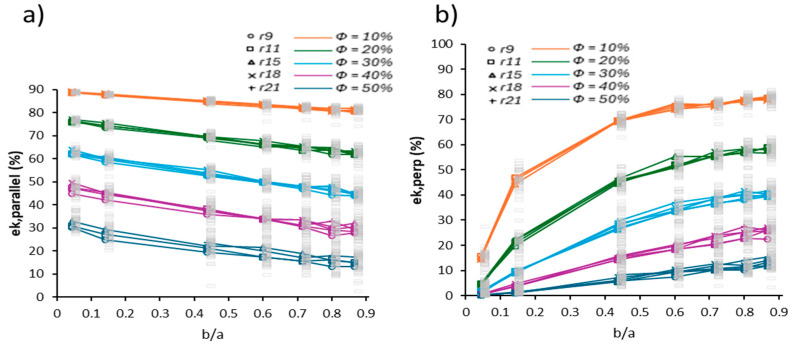
(**a**) $e_{k,parallel}$ as a function of the semi-axis ratio b/a for pores oriented parallel; (**b**) $e_{k,perp}$ as a function of the semi-axis ratio b/a for pores oriented perpendicular to the charge transport flow.

Each Ω configuration was evaluated using 10 independent stochastic realizations (ω_1_…ω_10_), generated with distinct random seeds. The standard deviation of the resulting $e_{k}$ distributions remained below 2–4% over the entire *εcc* range, confirming weak sensitivity to random initialization. This statistical stability is sufficient to characterize morphology-dependent transport trends, as the variation induced by pore orientation or eccentricity exceeds the sampling noise by more than an order of magnitude. Therefore, the domain-level responses reported here reflect intrinsic geometric effects rather than seed-dependent artifacts. Characterization of the membrane microstructures was carried out using a statistical approach. For each Ω configuration, 10 stochastic realizations were generated. The simulated configurations covered the ranges 10%≤ϕ≤50% and 0.5≤εcc≤0.999. In Figure 3, Figure 4 and Figure 5, gray dots correspond to individual ω realizations, while colored symbols represent the mean of each Ω average. Complete numerical data are available in Supplementary Table S1.

Figure 3 shows the evolution of the geometric descriptors. Figure 3a presents the analytical evolution of the b/a ratio as a function of εcc, which analytically makes strict sense with Equations (3) and (4). Figure 3b presents the trend of $E_{xy}$ (Equation (6)) with respect to εcc, and Figure 3c presents the trend of $E_{xy}$ (Equation (6)) with respect to b/a.

The observed decreasing trend was geometrically expected: as εcc approaches 1, the pore shape becomes more elongated; the semi-minor axis b decreases and the semi-major axis a increases, hence b/a→0. Figure 3b,c shows the $E_{xy}$ behavior, a structural anisotropy metric derived from the comparison between correlation functions in orthogonal directions. The results reveal that isotropy is conserved at $E_{xy}<0.002\;$up to a critical value near εcc=0.9, beyond which there is an exponential increase. This observation suggests the existence of a morphological transition in which the pore geometry begins to dominate the orientation of the porous medium, inducing significant directional anisotropy. This type of transition has been previously reported by Sadeghi et al. [29], who found that anisotropy in randomly generated porous membrane microstructures can emerge abruptly upon exceeding certain geometrical thresholds. Figure 3c shows that for the trend toward isotropy b/a→1 (εcc→0, the error $E_{xy}\to 0$). This behavior confirms the direct correlation between pore geometry and structural symmetry breaking of the medium.

The low dispersion observed among stochastic realizations demonstrates the robustness of the microstructure generation method. This stability is crucial for evaluating transport phenomena in anisotropic membranes, as variability associated with geometry must remain significantly below the effects induced by controlled distortion [36].

To isolate the intrinsic effect of eccentricity, perfectly aligned domains were selected. Real porous membranes feature orientation dispersion, distributing local pathways between parallel and perpendicular sub-domains and softening the contrast reported here. Intermediate angles yield partial conduction shortcuts that fall between the canonical limits, so the aligned cases represent bounding conditions: a best case for parallel transport and a worst case for transverse obstruction.

Figure 4 shows the evolution of $e_{k}\;$as a function of εcc, considering the two orientation configurations: parallel and perpendicular to the transport direction. The results are grouped according to porosity, and each curve represents the average Ω of the stochastic ω realizations.

The results in Figure 4 reveal a marked contrast between the parallel and perpendicular pore orientation configurations with respect to effective charge transport flow:

- In the parallel configuration, $e_{k,parallel}$ grows linearly and moderately with increasing eccentricity from de εcc=0.5 to εcc=0.999, with average values ranging from $e_{k,parallel}=80\%$ to $e_{k,parallel}=88\%$, for *Φ* = 10%. The difference between $e_{k,parallel}@\varepsilon cc=0.5$, and $e_{k,parallel}@\varepsilon cc=0.9$ is increasingly larger as the porosity increases. This trend suggests that pore elongation aligned with the charge gradient favors more direct and less tortuous conductive paths, which reduces the effective resistance of the medium. Similar results have been reported by Li et al. [37], who demonstrated that anisotropic functional membranes with pores aligned parallel to the flow maintain high relative conductivities, even under compressive conditions.
- In contrast, in the perpendicular orientation (Figure 4b), the efficiency $e_{k,perp}$ decreases progressively with increasing eccentricity and the difference between $e_{k,parallel}@\varepsilon cc=0.5$ and $e_{k,parallel}@\varepsilon cc=0.9$ becomes smaller and smaller (as porosity increases). This reduction responds to the increase in tortuosity and loss of transverse connectivity generated by elongated pores in the direction perpendicular to the charge transport flow. The charge-transport flow is forced to go around the pores, which increases the effective length of conductive paths and creates dead zones. Kang et al. [32] showed that such misalignment can cut effective transport coefficients by 40–70%, corroborating reductions in conductivity of up to 60% in anisotropic PEM membranes.

Figure 4 contains the analytical information necessary for the user of a microstructure, synthetic or experimental to determine $e_{k}\;$as a function of εcc. This can be conducted analytically by the image in Figure 4.

The transport degradation under perpendicular alignment can be interpreted in terms of tortuosity, defined as τ = L_eff_/L_dir_. Increasing εcc forces the conductive paths to circumvent elongated cavities, raising L_eff_ relative to the imposed direction L_dir_. In parallel alignment, pore elongation compresses local trajectories along the transport axis, decreasing τ and maintaining high connectivity. This metric provides a structural interpretation consistent with the observed $e_{k}$ trends.

Figure 5 shows the resulting plots of $e_{k}$ as a function of axis ratio (b/a). It is important to note that the values of $e_{k}$ presented in Figure 4 and Figure 5 are exactly the same, since both representations are derived from the same set of simulations. The difference lies only in the geometric parameter used as the independent variable. The b/a ratios can be determined analytically using Equations (3) and (4), which relate to the semi-axes b of the pores. This alternative presentation option has a practical purpose: it allows the user to estimate directly from a microstructural image (synthetic or experimental), simply by graphically measuring the lengths of the major and minor axes of the pores. In this way, a simple and intuitive route is provided to apply the results of this study to the analysis of real materials.

In realistic porous membranes, pores rarely exist as isolated entities, but rather form partially connected domains through necking, coalescence, and bridging. Connectivity modifies the effective length of conductive paths and introduces directional biases. Under parallel alignment, inter-pore bridges shorten the transport distance by generating longitudinal channels that bypass local constrictions. Conversely, under perpendicular alignment, connectivity amplifies bottleneck formation because transverse necking funnels carriers through narrow corridors, increasing tortuosity and path fragmentation. This dual role of connectivity has been reported in compressed PFSA and carbon-based membranes, where mechanical deformation promotes anisotropic pore merging and preferential conduction pathways [38].

These numerical results show that increasing the eccentricity εcc produces a significant decrease in the charge transport efficiency $e_{k}$ when pores are oriented perpendicularly to the transport direction, while this trend is much less pronounced for parallel alignment. Similarly, Sharma et al. [34] reported increases in polarization losses with perpendicular pore orientations. This contrast is consistent with the differences in overall tortuosity imposed by both configurations. Furthermore, Yao et al. [35] demonstrated that pore geometry significantly influences connectivity and transport efficiency. Ricci et al. [36] emphasized that the orientation and shape of the porous phase can critically impact flow distribution and transport efficiency in functional materials, particularly in electrochemical cells. Additionally, Zhao et al. [39] saw a similar impact of the porous structure on mechanical properties.

Quantitatively, the perpendicular configuration exhibited a reduction of 60–75% in $e_{k}$ for ϕ ≈ 10 % when εcc → 1. These values are consistent with conductivity losses reported for misaligned pores in 3D porous domains, where intermittent velocity structures decrease effective transport by 40–70% [36], and polarization penalties of ~55–65% are measured in microstructured cathodes [37]. The magnitude of the degradation therefore aligns with established experimental and computational evidence, even though the present model is restricted to two dimensions.

The aspect ratios explored here (b/a from 1.0 down to ~0.1) are comparable to the morphological distortions observed in compressed PFSA and carbon-based membranes, where originally circular voids collapse into elongated cavities under assembly pressure, humidity cycling, and operating load [38]. These deformation levels occur without altering bulk chemistry or pore creation mechanisms, and reflect geometric reshaping of existing voids. Accordingly, the eccentricities probed numerically fall within realistic morphological ranges while remaining independent of material degradation or fabrication artifacts.

Similarly, the results of Li et al. [37] demonstrate that geometrically aligned anisotropy favors the formation of preferential conduction pathways in diffusion layers with controlled orientation, thereby enhancing electrical conductivity. In contrast, when pores are oriented perpendicular to the applied electric field, the conduction efficiency $e_{k,perp}$ drops dramatically, often below 20% for specific values of εcc. This decline is attributed to increased transverse tortuosity and the formation of narrow bottlenecks that hinder connectivity. Liu et al. [40] further incorporated morphological penalty functions dependent on pore orientation and elongation, confirming that these parameters directly influence the design of functional porous membrane microstructures.

Pore size constitutes a secondary geometric variable that modulates the continuity of conductive pathways under constant porosity. When the void fraction is fixed, a reduction in the characteristic pore diameter increases the number density of pores, enlarges the total inter-pore interface, and promotes curvature of the local transport paths. This fragmentation effect amplifies tortuosity under perpendicular alignment because charge carriers must circumvent a higher frequency of insulating obstacles, generating bottleneck regions and longer effective transport distances. Similar mechanisms have been reported in stochastic porous media models, where morphological subdivision increases the effective resistive length despite preserving global ϕ [40]. In contrast, under parallel alignment, smaller pores distribute preferential conduction channels more uniformly, partially mitigating path curvature and stabilizing connectivity.

Although changes in the semi-major axis a and semi-minor axis b do not alter the overall porosity ϕ, the ratio b/a and its orientation with respect to the transport direction critically affect structural continuity and effective connectivity. In this context, the semi-major axis does not merely define the pore shape; it also acts as a key modulator of transport performance in anisotropic microstructures.

These findings highlight the importance of incorporating geometric descriptors (such as eccentricity, aspect ratio, and orientation) into computational membrane design.

The proposed finite-volume framework provides a predictive tool for optimizing pore architecture and maximizing membrane transport efficiency, making it suitable for rational design and inverse engineering of functional membranes.

The present computational domain is strictly two-dimensional; therefore, the effective conductivity values should be interpreted as relative descriptors rather than quantitative predictors for three-dimensional membranes. In real 3D structures, out-of-plane connectivity introduces additional conduction channels that reduce tortuosity, while volumetric percolation enables transverse bypasses around elongated pores both mechanisms increasing the absolute $e_{k}$ compared to 2D domains. Accordingly, the model isolates the intrinsic geometric influence of eccentricity and orientation on transport pathways, without attempting to approximate real 3D performance.

The contrast observed here, with improved connectivity under a parallel alignment and pronounced obstruction under a perpendicular alignment, originates from competition between continuous conducting lanes and the formation of geometric bottlenecks. These mechanisms are independent of dimensionality, although absolute conductivity values are expected to differ in realistic membranes. For this reason, the interpretation of the numerical results must be restricted to two-dimensional geometries. Although similar obstruction and channeling effects have been reported in 3D porous media, the additional dimensionality introduces transverse connectivity, out-of-plane pathways, and volumetric percolation that cannot be inferred from a purely 2D analysis. Therefore, any correspondence between the present 2D findings and realistic 3D membranes should be understood exclusively in terms of geometric trends, without implying quantitative agreement or predictive capability.

## 4. Conclusions

This work provides a controlled numerical assessment of how pore orientation, eccentricity (εcc), and semi-axis ratio (b/a) influence charge transport efficiency ($e_{k}$) in two-dimensional anisotropic porous membranes. The simulations show that aligning elongated pores parallel to the transport direction preserves continuous conduction pathways and maintains high $e_{k}$ values, while perpendicular alignment promotes bottleneck formation and transverse path diversion. For ϕ ≈ 10%, efficiency reductions of 60–75% were observed when εcc→ 1, reflecting the severe obstruction of conductive paths in this configuration.

The semi-axis ratio (b/a) modulates the severity of these trends. Under perpendicular alignment, higher *b*/*a* values mitigate the formation of narrow channels and partially restore connectivity, whereas under parallel alignment, similar elongations do not confer additional transport benefit. This dichotomy highlights that the impact of pore geometry is orientation dependent, and can reinforce or attenuate anisotropy in distinct ways.

These results establish quantitative structure–property relationships between geometric descriptors and macroscopic transport behavior within the adopted 2D framework. Because the model does not include out-of-plane connectivity, volumetric percolation, chemical interactions, or compression-induced changes in porosity, the reported $e_{k}$ values should be interpreted as relative indicators of geometric sensitivity rather than predictive metrics for real membranes.

Within this scope, the study clarifies the geometric conditions that favor or hinder conduction, and provides a baseline for analyzing anisotropy in porous membrane microstructures. The findings may inform future extensions to three-dimensional models and experimental validation, but do not imply direct transferability to operational membrane systems.

## Data Availability

The data that support the findings of this study are available from the corresponding author on reasonable request.

## Supplementary Materials

The following supporting information can be downloaded at https://www.mdpi.com/article/10.3390/membranes15120370/s1: Table S1, Complete simulation dataset; and File S1, Supplementary Material document providing detailed descriptions of the numerical dataset.
