# Supplementary material for: Numerical Assessment of Elliptical Pore Orientation and Eccentricity Effects on Charge Transport in Anisotropic Functional Membranes
Source: Membranes (Basel). 2025 Dec 2;15(12):370. doi: 10.3390/membranes15120370 (PMC12735207; doi:10.3390/membranes15120370)
Supplement: Supplementary file 1 [file membranes-15-00370-s001.zip › Supplementary Material.pdf]

Supplementary material

Manuscript title: Numerical Assessment of Elliptical Pore Orientation and Eccentricity Effects on Charge Transport in Anisotropic Functional Membranes

Authors: Carlos Pacheco, Alfonso Navarro, Enrique Escobedo and Romeli Barbosa*

Centro de Investigación Científica de Yucatán, Parque Científico y Tecnológico de Yucatán, Carretera Sierra Papacal–Chuburna Puerto, km 5, Sierra Papacal, Merida 97302, Yucatan, Mexico *Corresponding author: romeli.barbosa@cicy.mx or romelix1@gmail.com

Abstract
This supplementary material provides access to the complete simulation dataset and supporting computational tools used to analyze the influence of pore eccentricity and orientation on charge transport efficiency in anisotropic porous media. It includes numerical results (1652 configurations), statistical averages. The materials are intended to ensure reproducibility, transparency, and ease of comparison with future studies in porous material design.

Supplementary Table S1: Detailed simulation dataset and charge transport results.

The Excel file Supplementary_Table_S1.xlsx contains the complete dataset used in the simulations supporting Figures 3, 4, and 5 of the main manuscript. The file is organized into two sheets:

Sheet 1. Full simulation dataset: 10 random ω samples for each Ω configuration (used in Figure 3)

This sheet contains the raw simulation results for a total of 1650 microstructures. Each configuration is defined by a unique combination of geometric parameters Ω = { Φ, R, εcc}, and is evaluated across 10 independent random realizations ω to capture the statistical variability associated with stochastic pore distribution.

Each row corresponds to a unique microstructure instance. These data directly support the statistical dispersion analysis shown in Figure 3, illustrating the sensitivity of transport efficiency to pore orientation and eccentricity under varied mesostructural conditions.

Sheet 2. Averaged results (used in Figures 4 and 5)

This sheet presents mean values calculated over the 10 ω samples corresponding to each Ω condition. The summarized dataset provides the basis for the trend analyses and comparative plots in Figures 4 and 5, where systematic variations in eccentricity and porosity are explored for both parallel and perpendicular pore orientations.

Both Sheet 1 and Sheet 2 contain the following nine columns:

Column A. Φ, Porosity (volume fraction of voids).
Column B. R, Radius of a circular pore.
Column C. εcc, Pore eccentricity.
Column D. a, Semi-major axis of elliptical pore (Equation (3), manuscript).
Column E. b, Semi-minor axis (Equation (4), manuscript).

Column F. b/a, Aspect ratio of the pore.

Column G. E_{xy}, Normalized autocovariance XY error (Equation (6), manuscript)

Column H. $e_{k,parallel}$, Charge transport efficiency (numerical solution of Equation (7)), when the major axis is aligned parallel to the electric field.

Column I. $e_{k,perp}$, Charge transport efficiency (numerical solution of Equation (7)), when the major axis is oriented perpendicular to the field.

Together, these data ensure full transparency and reproducibility, and serve as a robust benchmark for future computational or experimental studies focused on charge transport phenomena in anisotropic porous media.
